# Autoimmune Encephalitis in a Girl With Psychiatric Manifestations

**DOI:** 10.7759/cureus.14512

**Published:** 2021-04-16

**Authors:** Anum Haider, Areeba Rehman, Summaiyya Waseem, Saad Khalid

**Affiliations:** 1 Psychiatry, Dr. Ruth K.M. Pfau Civil Hospital, Dow University of Health Sciences, Karachi, PAK; 2 Internal Medicine, Dow Medical College, Dow University of Health Sciences, Karachi, PAK; 3 Neurology, Dow Medical College, Dow University of Health Sciences, Karachi, PAK; 4 Surgery, Dow Medical College, Dow University of Health Sciences, Karachi, PAK

**Keywords:** autoimmune encephalitis, psychiatry, psychosis

## Abstract

Autoimmune encephalitis (AIE) can be caused by various neuronal surface and intracellular antibodies. The prevalence and gender predisposition vary according to the subtype involved. It can produce a wide range of neurologic or psychiatric symptoms. We present a case of a young female patient who presented to a psychiatric facility with behavioral and perceptual disturbances and was later referred to a medical department, where, through a series of investigations, she was diagnosed as a case of AIE. Unfortunately, the ultimate outcome was death due to a delay in reaching toward accurate diagnosis. This case report highlights the lapse of the healthcare system, particularly in low- and middle-income countries at multiple levels in providing adequate health care.

## Introduction

Autoimmune encephalitis (AIE) consists of a group of classified but rare diseases. This family of diseases is caused by autoantibodies targeted against several intracellular and extracellular neuronal proteins. These autoantibodies are often used as a biomarker and differentiate the several AIE subgroups [1].

AIE can manifest itself with isolated psychiatric symptoms or with initial psychiatric symptoms with a later expression of neurological ones with the dynamic phenotypic pattern [2,3]. For this reason, patients are often first admitted to the psychiatric ward for medical clarification, raising a subset of questions about patients whose etiologies are misdiagnosed due to availing inappropriate pathway of care [4]. In low- and middle-income countries like Pakistan, there is no standard system of referring patients through systemic channel in order to provide adequate and timely healthcare services. In our setup, generally patients adopt pathway of care depending on their own cultural and health beliefs.

Antineuronal antibodies are detectable in cerebrospinal fluid (CSF) of 54.5% of psychotic patients with autoimmune causes [5]. However, despite the availability of tests to confirm treatable disorders, they are inaccessible due to financial restraints and burden of patients producing hindrance in the localization of resources, both diagnostic and therapeutic.

We report a case of a young female patient who presented to the psychiatry department with behavioral and perceptual disturbances by making previous visits to faith healers and general physicians. She was ultimately referred to the neuromedicine department, where she was later diagnosed with AIE. This report is intended to raise the attention of stakeholders and policymakers to develop an accessible referring healthcare system for the general public to provide a standard pathway of care.

## Case presentation

A 13-year-old school-going girl, inhabitant of Karachi, was brought to an outpatient psychiatric facility at a tertiary care public sector hospital in Karachi, Pakistan, by her family in December 2019, with acute onset of behavioral disturbance about two months ago that consisted of fearfulness and suspiciousness as someone stalking her or planning to kill her. She was also seeing some faces that she had difficulty differentiating from dreams. She lost focus in her studies and felt uncomfortable when socializing. She had low-grade fever preceding those features but got settled easily. During those two months, her family initially sought spiritual treatment from the faith healers and then consulted a general physician who prescribed antipsychotic risperidone 2 mg, but she could not tolerate it due to tremors and other side effects that appeared within first 24 hours and progressed gradually. Hence, on the fourth day of risperidone use, she was switched to another second-generation antipsychotic, olanzapine 5 mg, on which she partially responded by alleviation in agitation and irritation, but within a week, tremors and rigidity again appeared. At that time, she was referred by another physician to a psychiatrist and consequently visited us. On assessment, she appeared anxious, screaming with fear, perceiving the environment as threatening and insecure. Her cognitive functions seemed unremarkable grossly. There was no psychiatric disorder in the family; neither patient had any psychiatric or medical problem before. The school environment was also reported to be secure and friendly, and there was also no account of any trauma or abuse. We tapered off her olanzapine dose and referred her to the neuromedicine department considering the peculiar features, including young age, appearance of side effects even at a lower dose of antipsychotics, and the presentation of psychiatric features of visual hallucinations, perplexed, and anxious look of the patient, which were more suggestive of an organic disorder.

The family approached a private medical facility on outpatient basis, where general biological investigation was advised, including complete blood count, liver and thyroid function tests, urea, creatinine and electrolytes, and viral markers, and all came out to be in a normal range. But within a week, her condition deteriorated and she developed an altered level of consciousness. The family brought her to a tertiary care hospital for admission. She was investigated first for neuroleptic malignant syndrome, but that did not explain her state. During hospitalization, she had seizure episodes and was investigated immediately and managed in line with encephalitis. Her CSF was sent for immunoanalysis when antinuclear antibody profile turned out positive. The family and doctors made arrangements for immunotherapy, keeping both options of intravenous immunoglobulins (IVIGs) and plasmapheresis available, but the young girl could not survive and died the following week.

## Discussion

AIE consists of a set of syndromes that are characterized by limbic and extra-limbic disorder manifestations, occurring due to the presence of antibodies. When broadly classified, two categories of antibodies cause AIE, which are neuronal surface and intracellular antibodies [6,7]. The most common neuronal autoantibody detected is the anti-N-methyl-D-aspartate receptor (NMDAr) antibody, and the most common intracellular antibody is the anti-Ri antibody [7]. Other autoantibodies in the neuronal surface group consist of α-amino-3-hydroxy-5-methyl-4-isoxazolepropionic acid receptor, contactin-associated protein-2, large glucagon immunoreactivity-1 (LGI-1) and gamma-aminobutyric acid receptor antibody [8,9].

AIE was once considered rare, but data have shown an increase in their prevalence over the years. According to a study conducted in the USA, the incidence of this disease has amplified from 0.4/100,000 person-years (1995-2005) to 1.2/100,000 person-years (2006-2015) due to increased recognition of neural-specific IgG-associated encephalitis [10]. AIE differs according to the subtype involved, with anti-NMDA much more prevalent in women of a certain age group, particularly 12-45 years. However, in some subtypes, more cases are seen in men: anti-LGI1 and CASPR2 AE [11].

Patients with AIE present with variable neurologic and psychiatric or initial flu-like symptoms and fever as occurred in our case. Neurologic symptoms of NMDAr antibody encephalitis may consist of dementia and cognitive dysfunction, atypical movements, seizures, ataxia, and speech or vision problems [12]. Psychiatric manifestations, such as psychosis, violence, inappropriate sexual behaviors, panic disorder, compulsive behaviors, euphoria, and anxiety, can also be seen along with neurological symptoms or may be the only presenting feature [12] as in our case and can lead to misdiagnosis, mismanagement, and ultimately worsening of prognosis.

The exact mechanism of neuropsychiatric symptoms like the ones in our case (behavioral disturbances) is still unknown. However, it is speculated that NMDAr antibodies may cause inhibition in presynaptic GABAergic interneurons, leading to reduction in GABA release. This results in the disinhibition of postsynaptic glutamatergic transmission, leading to glutamate-dopamine dysregulation [13].

Symptoms in AIE can lead to syncope and can even be fatal, if not detected early, as seen in our case [12]. Since this disease presents with a spectrum of clinical presentations and symptoms, diagnosis is often delayed, leading to worsening of the condition and increased mortality in these cases. The reported mortality rate of AIE is around 8%-18.45% even in developed countries [14]. 

For the detection of AIE, immunoblotting, immunohistochemistry, and immunocytochemistry with immunofluorescence assays are most often used [7].

First-line therapy for AIE includes IV steroids, IVIG, and plasmapheresis. For patients refractory to first-line therapy and not showing improvement within two weeks, treatment with second-line agents, such as rituximab and cyclophosphamide, is recommended [15]. Second-generation antipsychotic medications, such as olanzapine, can manage agitation and aggression in anti-NMDAR encephalitis but there is little improvement in psychosis, affective symptoms, or catatonia with antipsychotics alone [11]. Our patient also got subtle response with it and developed side effects later on. Antipsychotic medications usually have a number of adverse effects including poor tolerability to drug, mild sedation, dry mouth, constipation, sexual dysfunction, akathisia, dystonia, and tardive dyskinesia. It should be given to patients after proper assessment because patients with organic disorders are more prone to these side effects [16].

Keeping lapse in the present healthcare system aside, with the expectation of improvement by policymakers in the times to come, the healthcare practitioners specifically associated with neuropsychiatric specialties must show vigilance in identification of treatable organic conditions at the earliest possible time, especially in those cases where symptom profile and sociodemographic characteristics do not support the typical psychiatric disorder.

## Conclusions

AIE is a treatable disease, and timely diagnosis is imperative to improve outcomes and decrease mortality. Early treatment can reduce long-term complications, rapid rehabilitation, and less likelihood of AIE relapses. It is important to differentiate organic possibilities first in each patient where clinical presentation and response to treatment are variable. The establishment of standard referring healthcare system is crucial in order to improve quality of care, patient prognosis, and outcome.
